# An endothelial growth factor receptor compound mutation of T790M substitution with exon 19 deletion in a previously untreated patient: a case report

**DOI:** 10.1186/s13256-019-2075-y

**Published:** 2019-05-15

**Authors:** Juan C. Falla-Martinez, Daniela Espinosa, Juan C. Baena, Lisa X. Rodriguez, Luz F. Sua, Angela R. Zambrano

**Affiliations:** 1 Hematology-Oncology department, Fundacion Valle del Lili, Carrera 98 No. 18-49, Fundacion Valle del Lili, Cali, Colombia; 2 Internal Medicine department, Fundacion Valle del Lili, Carrera 98 No. 18-49, Cali, Colombia; 3 Human Genetics department, Fundacion Valle del Lili, Carrera 98 No. 18-49, Cali, Colombia; 4 Pathology department, Fundacion Valle del Lili, Carrera 98 No. 18-49, Cali, Colombia

**Keywords:** Lung neoplasms, EGFR protein, Adenocarcinoma of the lung, Compound mutation

## Abstract

**Background:**

Endothelial growth factor receptor (*EGFR*) mutations are an essential driver of personalized therapy for patients with lung cancer and are detected in approximately 15% of Caucasian and 50% of Asian patients. *EGFR* tyrosine kinase inhibitors have been developed and used for this set of patients. T790M mutation in exon 20 is usually associated with secondary resistance to *EGFR* tyrosine kinase inhibitors therapy but is also present in treatment-naïve patients. The frequency for baseline T790M mutation varies from 4 to 35% according to the detection method used. Newer techniques have yielded higher rates, but concerns about false-positive results have been raised. Compound mutations account for 4–14% of all *EGFR*-mutated tumors, with no studies yet to provide a frequency rate for T790M + 19 deletion association due to the small number of cases. However, there are reports that pretreatment T790M + L858R association is significantly more frequent compared to T790M + exon 19 deletion mutations. Diagnostic challenges, current knowledge on the subject, and therapeutic decisions are discussed.

**Case presentation:**

We present the case of a 43-year-old Hispanic woman, a treatment-naïve patient, with metastasized lung cancer adenocarcinoma harboring a T790M deletion along with the classic 19 mutation. The initial symptoms were monoparesis of her left leg, associated with hyperreflexia, and hypoesthesia. In the absence of third-generation tyrosine kinase inhibitors, a platinum-based therapy was initiated with no response and she died 4 months after diagnosis.

**Conclusions:**

Osimertinib seems to be a suitable therapy for treatment-naïve patients with sensitizing and resistant compound *EGFR* mutations. More studies regarding the clinical characteristics of these patients and the appropriate management of this condition are needed to provide the highest standard of care.

## Background

Non-small cell lung cancer (NSCLC) accounts for the majority of lung neoplasms and its mortality in advanced stages is very high, compared with other cancer types [1, 2]. Driver mutations can be found in a high percentage of these cases, including endothelial growth factor receptor (*EGFR*) alterations [3–5]. However, compound mutations in treatment-naïve patients are rare, even more so if the association is that of a T790M mutation and an exon 19 deletion (19-Del) [5, 6]. There are very few cases of this compound mutation found in the literature.

We present the case of a 43-year-old, treatment-naïve, woman with metastasized lung cancer adenocarcinoma harboring a T790M deletion along with the classic 19-Del. The importance of this case lies in the rareness of this compound mutation, the ample variation of T790M incidence in the literature, and the multiple treatment difficulties that arise when, for a particular reason, including no availability, a third-generation *EGFR* tyrosine kinase inhibitor (TKI) cannot be used.

## Case presentation

A 43-year-old Hispanic woman with no past medical or family history of importance, presented to our emergency room (ER) with progressive lumbar pain for the past 4 months, 10/10 in intensity, which irradiated to her left lower limb limiting her functionality. She also referred weakness of her left leg, associated with loss of sensitivity, and had experienced night fevers, chills, and a 23 kg (50 pound) weight loss. A physical examination revealed monoparesis of her left leg, associated with hyperreflexia, and hypoesthesia.

**Fig. 1:**
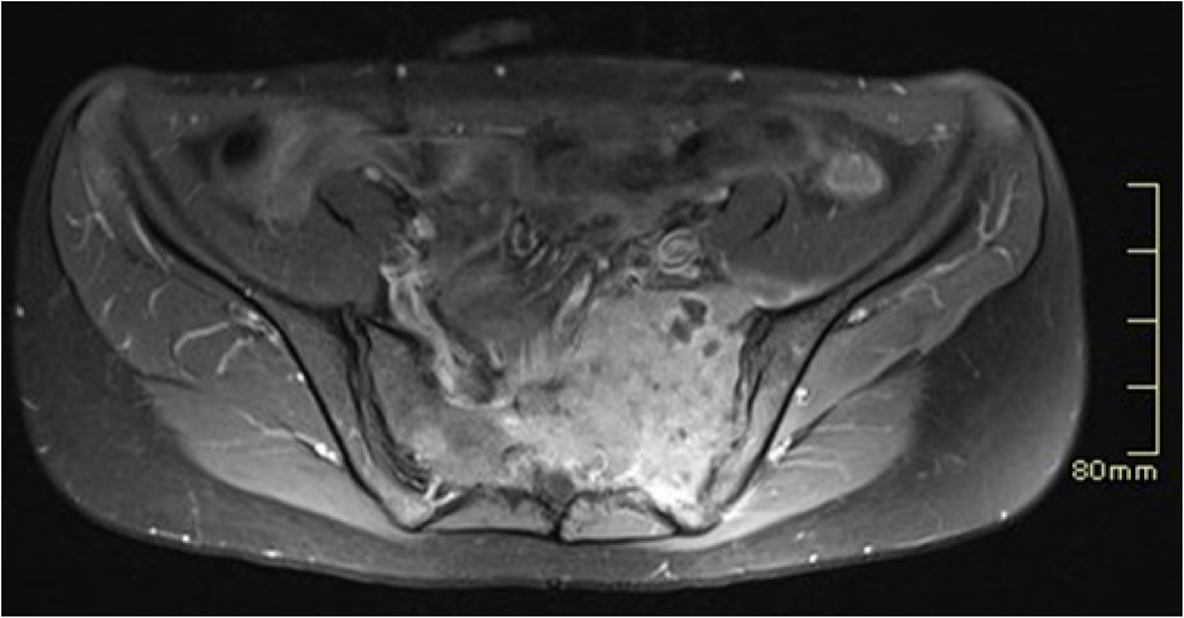
Pelvic magnetic resonance imaging. Infiltrative mass with areas of cystic appearance in the left sacral bone, extending to the sacroiliac joint and left iliac bone, obliterating the left neural foramina. Measures 6.2 × 7.9 × 5.3 cm

A contrasted pelvis and lumbar magnetic resonance imaging (MRI) showed a solid infiltrative mass in her left sacral and iliac bones, compromising the left sacroiliac joint, the ipsilateral sacral nerve roots, and the pyramidalis and gluteus medius muscles. Other bone lesions compromised the left femoral neck and the right femoral diaphysis (Fig. 1).

**Fig. 2:**
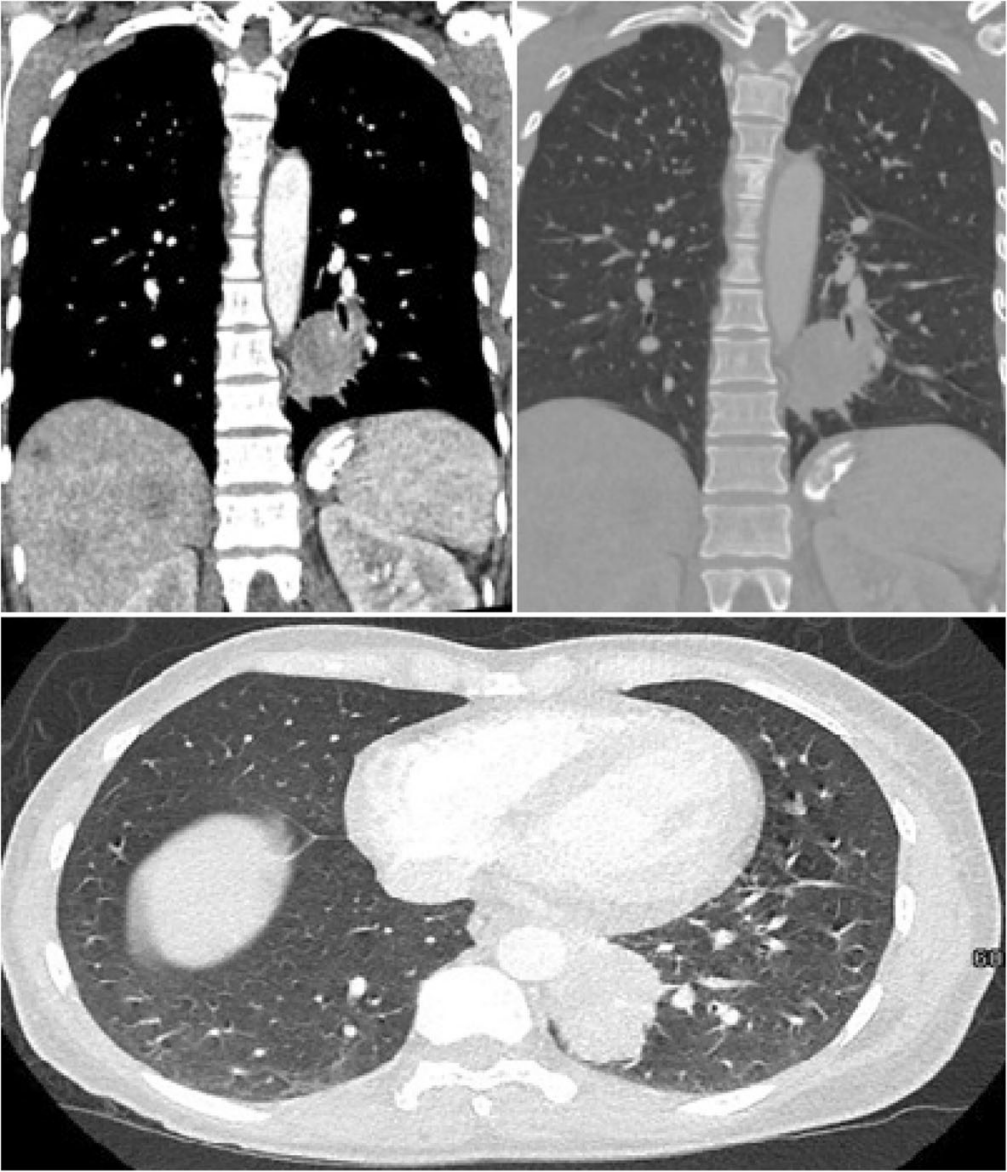
Thorax computed tomography. Solid mass with heterogenic enhancement, with well-defined intrapulmonary spiculated contours in the anteromedial segment of the lower lobe of the left lung of 4.7 × 3.3 cm (anteroposterior × transverse)

**Fig. 3:**
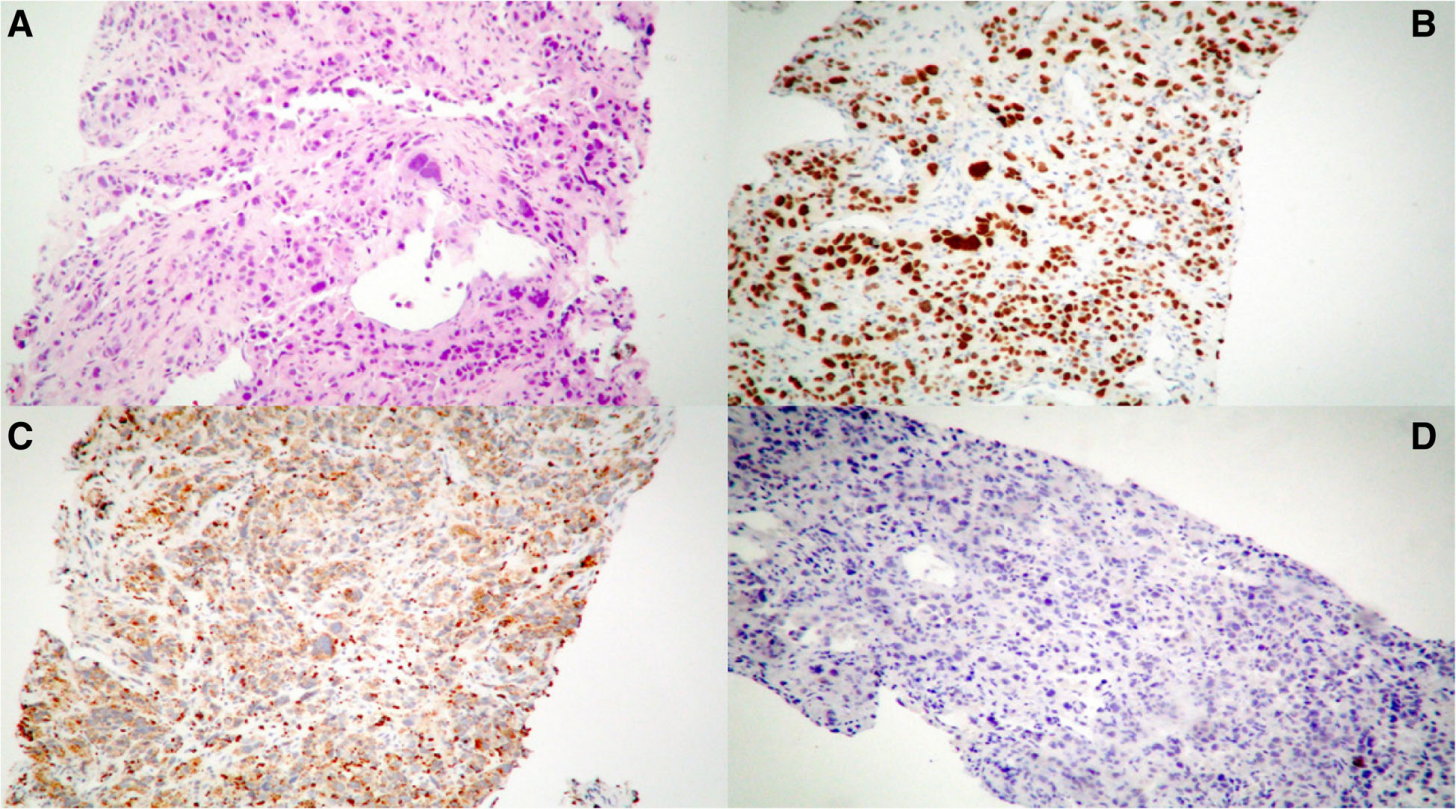
Metastasized lung adenocarcinoma in sacral bone. Sacral bone biopsy. **a** Hematoxylin and eosin stain, solid pattern metastatic adenocarcinoma of the lung (× 10). **b**, **c** Positive thyroid transcription factor 1 and napsin A immunohistochemistry stain (× 10). **d** Negative immunohistochemistry stain for EML-4, ALK, and programmed death-ligand 1 rearrangements

The hypothesis was that these lesions were metastatic, so further studies were ordered. Breast ultrasonography revealed a mass of 2 cm by 3 cm in her left breast, but a subsequent fine-needle biopsy showed benign histopathology. A computed tomography (CT) scan revealed masses in both her liver and lung (Fig. 2). A bronchoalveolar wash was negative for malignancy, and so was a transbronchial biopsy. A decision was made to do a CT-guided percutaneous biopsy of the sacral lesion; the results revealed a metastasized lung adenocarcinoma (Fig. 3), negative for ALK mutation but with a complex mutation of the *EGFR* gene: a 19-Del associated with a T790M (exon 20) mutation. The genetic assay used was cobas® EGFR Mutation Test v2 (Roche®). The target deoxyribonucleic acid (DNA) was amplified and detected on the cobas® 480 system which measures the fluorescence generated by specific polymerase chain reaction (PCR) products, using the amplification and detection reagents provided in the cobas® EGFR mutation test kit (lightmix®).

Stage IV lung adenocarcinoma was diagnosed. In the absence of third-generation *EGFR*-TKIs (not approved by the local drug and food administration at the time) and taking into account the performance status of our patient, which was Eastern Cooperative Oncology Group (ECOG) 2, platinum-based chemotherapy of gemcitabine (1000 mg/m^2^ day 1 and 8) with carboplatin (AUC 6) was initiated. Only grade 1 toxicities were observed.

**Fig. 4:**
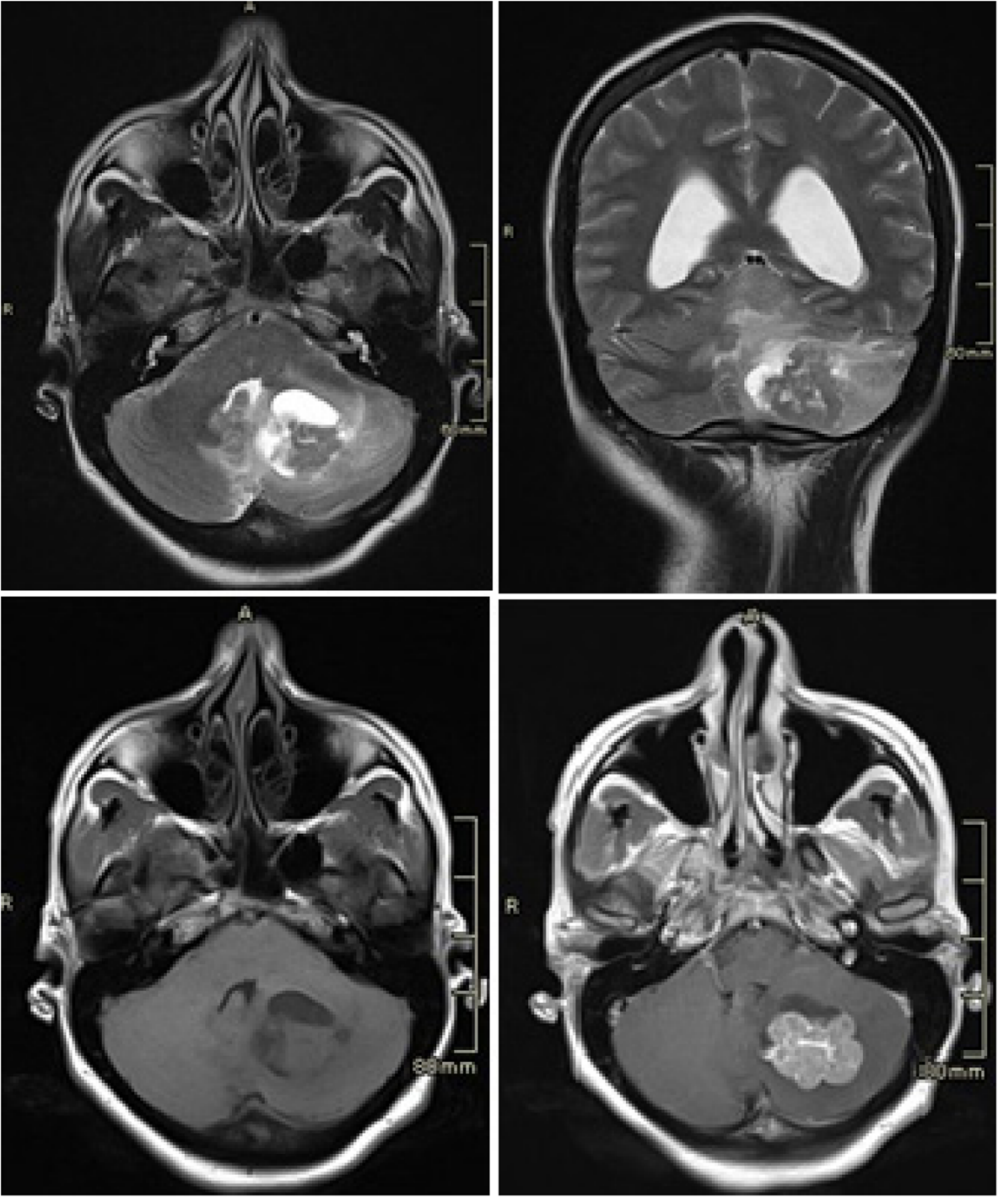
Brain magnetic resonance imaging. A nodular lesion with a cystic component of intra-axial location in the left cerebellar hemisphere is observed, whose enhancement component measures 23 × 31 mm (anteroposterior × transverse), is associated with vasogenic edema in the periphery and conditions the mass effect

Our patient suffered from a severe headache 25 days after admission, with no response to analgesia, and a CT scan of her brain was done; the CT scan revealed intraparenchymal bleeding on the left cerebellar hemisphere, with fourth ventricle compression and non-communicant acute hydrocephalus. A brain MRI showed a lesion in the cerebellum, thought to be a metastasis (Fig. 4). After stabilization of the clinical status, whole brain irradiation with three-dimensional conformational radiotherapy (CRT) was done (3 Gy fractions for a total dose of 30 Gy). Radiation therapy (three-dimensional CRT) was also administered to her left sacral and iliac bones for pain management (4 Gy fractions for a total dose of 20 Gy). She died 4 months after diagnosis.

## Discussion

NSCLC accounts for nearly 85% of all lung neoplasms, and its 5-year survival rate is still low, around 17% dropping to 5%, if the disease is categorized as stage IV [1, 2]. As many as 80% of cases of lung adenocarcinoma have a driver mutation contributing to early carcinogenesis [7]; the identification of these mutations has allowed for a more personalized therapy, improving outcomes in relevant populations. *EGFR* mutations, detected in approximately 15% of Caucasian and 50% of Asian patients [3, 4], have been targeted since 2004, resulting in *EGFR*-TKIs such as erlotinib and gefitinib.

*EGFR* mutations are more frequent in tumors with adenocarcinoma histology, in never-smokers or light smokers of tobacco, in women with NSCLC, and in patients with East Asian ethnicities [8]. The two most common *EGFR* mutations, also called classic mutations, are the 19-Del and the L858R substitution in exon 21 [9], both of them regarded as positive predictive biomarkers for response to *EGFR*-TKIs [10]. These mutations are oncogenic because they favor the active state of the kinase, inducing EGFR-mediated antiapoptotic and prosurvival proteins specifically in the Akt/STAT and MAPK signaling pathways [11].

T790M mutation in exon 20 is usually associated with secondary resistance to *EGFR*-TKIs therapy, being responsible for approximately 50 to 60% of these cases [12, 13]. This point mutation increases the affinity of *EGFR* for adenosine triphosphate (ATP) and decreases drug binding through steric hindrance, consequently diminishing the binding efficacy of *EGFR*-TKIs [12, 14–16]. If this mutation is present before treatment, then it becomes a rare *EGFR* mutation. The frequency of this mutation in treatment-naïve patients varies significantly according to the population screened and the method used for detection. The rate fluctuates between 1 and 3% when direct sequencing is used [16]. However, when newer techniques like real-time (RT) PCR, next-generation sequencing (NGS), and highly sensitive matrix-assisted laser desorption/ionization time-of-flight mass spectrometry (MALDI-TOF MS) are used, the frequency rises to 25 to 35% [17, 18]. One study reported a rate of 79% using colony hybridization [19]. However, there are reports of false-positive results with the newer techniques, especially when the sample tested is formalin-fixed paraffin-embedded tumor tissue [20–22].

Even rarer than an uncommon mutation is a compound mutation. This entails a dual mutation on the *EGFR* gene, comprising a sensitizing mutation (usually 19-Del or a 21 substitution) along with a rare mutation involving other residues of the tyrosine kinase domain of *EGFR* [5]. In our case, the double mutation was found to be a coexistence of 19-Del and the exon 20 T790M mutation. Compound mutations account for 4–14% of all *EGFR*-mutated tumors [5, 23, 24]. This frequency variation is due most likely to the fact that multiple methods for mutation detection are used among the studies. One article has independently associated the T790M and drug-sensitive *EGFR* mutations, establishing the hypothesis that these mutations themselves cause genetic instability, predisposing the cell to more DNA changes [25]. However, there seem to be some combinations that occur more often than others. Some studies have concluded that pretreatment T790M point mutation is significantly more frequent in patients carrying the L858R mutation compared to those harboring deletions in exon 19 [6], making this case even more unusual. Very few case reports of this compound mutation are published. Sakashita *et al.* [26] presented three cases of *EGFR* compound mutation, one of them a 19-Del + T790M alteration; their approach was a pathological one. Lou *et al.* [27] published one similar case but with no clinical description, and Khan *et al*. published a short report about a 57-year woman harboring the same mutation, with a good response to osimertinib [28]. A summary of cases reported in descriptive studies is presented in Table 1.

**Table 1:** Cases of exon 19 deletion + T790M in the literature

Author	Year	Type of assay	Number of samples	Exon 19 deletion + T790M
Yu *et al*. [29]	2018	ARMS-PCR	3925	43 (1.09%)
Illei *et al*. [30]	2017	NGS	1006	1 (0.09%)
Tezel *et al*. [31]	2017	PCR	959	2 (0.2%)
Tu *et al*. [32]	2017	ARMS-PCR / Sanger	5363	11 (0.2%)
Tang *et al*. [33]	2016	ARMS-PCR	3894	8 (0.2%)

*ARMS-PCR* amplification refractory mutation system technology, *NGS* next-generation sequencing, *PCR* polymerase chain reaction

There is a paucity of literature about the clinical profile of patients with *de novo* T790M mutation. Studies in Korea and Japan [34, 35] have described characteristics that are similar to those of patients with an *EGFR* mutation, regardless of the mutation: female gender, < 65 years of age, never-smoker of tobacco, and adenocarcinoma histology. The differences found were mainly a higher never-smoker of tobacco representation and more brain metastasis. All of these characteristics, including brain metastasis, were present in our patient.

Despite some studies concluding that first-line TKI therapy could benefit patients with a compound mutation harboring a T790M substitution [19, 36], the majority of the literature suggests that compound *de novo* T790M mutations confer resistance to TKI therapy. Poorer response rates to first-generation and second-generation *EGFR*-TKIs, along with similar response rates to platinum-based chemotherapy, have been described in this population. However, even though these patients have worse progression-free survival (PFS), there is no statistically significant difference in overall survival (OS) [17, 18, 32, 34–37]. New therapeutic molecules called third-generation *EGFR*-TKIs could be the solution for this set of patients, namely osimertinib.

Osimertinib is an orally administered, irreversible *EGFR*-TKI that is selective for activating *EGFR* mutations including 19-Del, L858R in exon 21, as well as the common T790M gatekeeper mutation mediating acquired resistance to early generation *EGFR*-TKIs [38]. Its potency is almost 200-times greater against L858R/T790M than the wild-type *EGFR*, which minimizes toxicity in patients [39].

Its clinical efficacy has been shown in multiple international trials, namely the AURA1 [40], AURA2 [41], AURA3 [42], and FLAURA [43] trials. The international phase I/II AURA clinical trial (NCT01802632) included patients with locally advanced or metastatic NSCLC with documented *EGFR* mutation or prior benefit to *EGFR*-targeted therapy following progression on at least one prior *EGFR*-TKI. The AURA phase II extension study established the safety and efficacy of osimertinib 80 mg daily as either second-line or third-line therapy in patients with *EGFR*-mutated NSCLC with confirmed T790M mutation. A pooled study of both the AURA1 extension and the AURA2 revealed a median duration of osimertinib treatment to be 16.4 months, with an improvement in the overall response rate (ORR) which was 66% (262 of 398 patients; 95% CI, 61–70%) and PFS [44]. Of note, there was a trend toward an increased response rate in patients who had co-occurring *EGFR* T790M mutations with 19-Dels versus *EGFR* T790M mutations with leucine-to-arginine at codon 858 (L858R) mutations (70 versus 57%) [44].

The AURA3 trial was a randomized international phase III study comparing osimertinib to platinum-pemetrexed chemotherapy combination in patients with T790M-mutated NSCLC following progression on prior *EGFR*-TKIs [42]. Osimertinib was superior, with a higher ORR (71 versus 31%; *p* < 0.001) and improved median PFS (10.1 versus 4.4 months, hazard ratio 0.30; 95% CI, 0.23–0.41; *p* < 0.0001) [42]. Nowadays, upon availability, osimertinib is the therapy of choice for this set of patients.

The FLAURA phase III, double-blinded, international clinical trial compared osimertinib to either gefitinib or erlotinib as initial therapy for patients with advanced *EGFR*-mutated NSCLC [43]. Longer median PFS was achieved in the osimertinib compared with treatment with standard-of-care first-line *EGFR*-TKIs (18.9 versus 10.2 months, hazard ratio, 0.46; 95% CI, 0.37 to 0.57; *p* < 0.001). However, no significant difference in the ORR was seen (80 versus 76%), but the median duration of response was longer with osimertinib (17.2 months) over standard *EGFR*-TKIs (8.5 months) [43]. Just as in the previous trials, patients with central nervous system (CNS) metastases had a better response to osimertinib. A study evaluating post-progression outcomes with first-line osimertinib versus standard-of-care *EGFR*-TKIs, provided further confidence in the interim OS data of the FLAURA study and advocated use of osimertinib as first-line therapy for *EGFR* mutant (*EGFR*m) advanced NSCLC [45]. The ELIOS trial (NCT03239340), which is ongoing, will assess the tumor genetic and proteomic markers at the point of disease progression in patients with *EGFR*m NSCLC who receive first-line osimertinib, hopefully providing better guidelines for the use of this drug as first-line treatment. However, further studies comparing second-generation *EGFR*-TKIs and osimertinib, and a more mature OS data for osimertinib, are needed to make a final decision [46, 47].

Very few data are available regarding the effectiveness of osimertinib in compound mutated *EGFR* NSCLC, at least in treatment-naïve patients with T790M/19-Del mutated tumors. Zhang *et al.* stated that first-generation TKIs, including gefitinib, erlotinib, and icotinib, are ineffective even with the presence of sensitive mutations when accompanied by a resistant mutation, while osimertinib carries survival benefits for those patients [48]. Yu *et al*. found a PFS of only 1.5 months for patients with sensitive and resistant compound mutation treated with first-generation and second-generation *EGFR*-TKIs [49]. Similar results were also observed in another prospective study, where the ORR and PFS were only 14.3% and 2.9 months, respectively for this set of patients [50]. With these results in mind, using osimertinib as a first line of treatment for patients with NSCLC harboring *de novo* compound T790M/19-Del mutation, like the one presented in this report, appears to be a sensible decision.

A choice for platinum-based chemotherapy was made for this patient due to the unavailability of third-generation TKIs, a baseline T790M mutation, the presence of multiple metastases, and a high tumor burden.

## Conclusion

Even though the rate of baseline T790M mutation is apparently higher than previously known, compound mutations harboring this point substitution are very rare, more so when accompanied by a 19-Del, making this set of patients particularly hard to manage. Osimertinib seems a good choice of treatment for these patients, but in its absence, and until more mature OS data are available and more comparative studies are made, first-line treatment can continue to be platinum-based chemotherapy, especially if there is a high tumor burden. More studies regarding the clinical characteristics of these patients and the appropriate management of this condition are needed to provide the highest standard of care.
